# A 1-year case-control study in patients with rheumatoid arthritis indicates prevention of loss of bone mineral density in both responders and nonresponders to infliximab

**DOI:** 10.1186/ar2219

**Published:** 2007-06-27

**Authors:** Hubert Marotte, Beatrice Pallot-Prades, Laurent Grange, Philippe Gaudin, Christian Alexandre, Pierre Miossec

**Affiliations:** 1 Hospices Civils de Lyon-bioMérieux Research Unit on Rheumatoid Arthritis, Hopital Edouard Herriot, place d'Arsonval, 69437 Lyon Cedex 03, France; 2 Department of Rheumatology, University Hospital, Hopital Bellevue, 42000 Saint-Etienne, France; 3 Department of Rheumatology, University Hospital, Hopital Sud, 38000 Grenoble, France

## Abstract

The goal of the present study was to record changes in bone mineral density (BMD) and markers of bone turnover in patients with rheumatoid arthritis (RA) who were treated with methotrexate combined (or not combined) with infliximab. Included were 90 patients with RA who required anti-TNF-α therapy with infliximab because of persistent active disease despite treatment with methotrexate. The historical control group included 99 patients with RA who were treated with methotrexate at a time when anti-TNF-α treatment was not yet available. Lumbar and femoral neck BMD was measured using dual energy X-ray absorptiometry at baseline and 1 year later. Osteocalcin, C-terminal cross-linked telopeptide of type I collagen, parathyroid hormone and 25-hydroxycholecalciferol were measured in plasma at baseline and 1 year later. At 1 year BMD had decreased in the control group at spine (*P *< 0.01) and femoral neck (*P *< 0.001). In contrast, BMD at spine and femoral neck did not change after 1 year of infliximab treatment. At the same time point, no change in bone remodelling markers was observed. No association was observed between clinical response and changes in BMD, indicating that even those who did not respond clinically did not lose bone over a 1-year period. These data confirm the BMD decrease observed in RA patients treated with methotrexate alone. This bone loss was prevented by infliximab therapy. Importantly, this beneficial effect was also observed in apparent nonresponders.

## Introduction

Rheumatoid arthritis (RA) is a chronic disease that is characterized by joint inflammation and local bone erosion. In addition, generalized bone loss has been demonstrated in RA patients [1,2]. This could be due to the disease itself, to reduced exercise activity, or to treatment with steroids [3], but it could also result from common postmenopausal osteoporosis.

Among the factors that can influence bone resorption and osteoclast activity, tumour necrosis factor (TNF)-α plays a central role in the destructive process of RA and has been shown to increase bone resorption in systemic osteoporosis related to oestrogen deficiency [4]. In transgenic mice expressing soluble TNF receptor to neutralize TNF-α, animals were protected from oestrogen deficiency-related bone loss [5]. TNF-α is also a powerful inhibitor of bone formation [6].

Infliximab is a neutralizing chimeric monoclonal anti-TNF-α antibody that has been successfully used in RA treatment [7], and has an effect on joint destruction [8]. However, its systemic effect on bone remains to be elucidated. In this study, we compared bone mineral density (BMD) values between RA patients treated with infliximab and those not receiving infliximab. Previous open studies have demonstrated either an increase in BMD or no change. A major limitation of these studies is that they did not include a control group [9,10]. The optimal design for this type of study would be a double-blind randomized comparison with placebo. However, because TNF-α blockers are now on the market, ethical issues would prevent such a randomized, placebo-controlled trial. Another option is to perform a historical control study, with controls being active RA patients followed before the advent of TNF-α blocker therapy and who were treated with methotrexate alone. Such a historical control group is of great interest because it is not influenced by use of TNF inhibitors in patients with the most active disease.

This case-control study was conducted to compare changes in BMD between RA patients treated with infliximab and those not receiving this agent over 1 year. Moreover, we investigated bone turnover using biochemical markers of bone formation and resorption, and we studied the relationship between changes in BMD and clinical response to therapy.

## Materials and methods

### Patients

All patients fulfilled the American College of Rheumatology criteria for RA [11] and gave informed consent to participate in this study, which was approved by the ethics committee. This study was performed by the investigators, independent of and unsupported by Centocor or Schering-Plough.

The control group included 99 patients (21 men and 78 women) who were consecutively enrolled before the advent of anti-TNF-α treatment, from 1996 to 2000. All of them were receiving methotrexate. The infliximab-treated group included 90 patients (16 men and 74 women) requiring anti-TNF-α therapy for treatment of persistent active disease, despite treatment with methotrexate. Patients were enrolled, starting from when infliximab entered the market, from January 2001 to October 2003. Infliximab was administrated at 3 mg/kg on weeks 0, 2 and 6, and then every 8 weeks combined with methotrexate (in accordance with the ATTRACT [Anti-TNF Therapy in RA with Concomitant Therapy] protocol [7]). All of these patients were included in the study and followed over 1 year.

RA activity was evaluated using the Disease Activity Score (DAS)28 [12], and a good clinical response was defined as an improvement of at least 1.2 in DAS28 score at 1 year.

### Bone mineral density evaluation

At baseline and 1 year later, BMD (g/cm^2^) was determined at the lumbar spine (first to fourth vertebrae, antero-posterior view) and at the right femoral neck, by dual-energy X ray absorptiometry using a QDR 4500 device (Hologic, Waltham, MA, USA). Quality control for the device was performed by daily assessment of a spine phantom. The *in vivo *precision error for dual-energy X ray absorptiometry, expressed as a coefficient of variation, was 0.9% at the lumbar spine and 1% at the femoral neck.

T scores (number of standard deviations [SDs]) from control individuals were calculated, in accordance with published reference values obtained in sex-matched control individuals studied using the same equipment at the same institution [13]. Osteopenia was defined as a T score between -1 and -2.5 SD and osteoporosis as a T score of -2.5 SD or less.

### Markers of bone turnover

All bone markers were measured using commercial assays, in accordance with instructions of the manufacturers. Osteocalcin (normal values 15 to 46 ng/ml), C-terminal cross-linking telopeptide of type I collagen (CTX-I; 330 to 782 pg/ml) and parathyroid hormone (PTH; 15 to 65 pg/ml) were measured using an Elecsys 2010 (Roche Diagnostics, Mannheim, Germany), and 25-hydroxycholecalciferol (25-OHD; 12 to 40 ng/ml) was measured using a radioimmunoassay (Incstar, Stillwater, MN, USA).

### Statistical analysis

Changes were compared between values at entry and at 1 year for BMD, DAS28, osteocalcin, CTX-I, PTH, 25-OHD, erythrocyte sedimentation rate and C-reactive protein. Data for both groups were compared using the unpaired Student's *t*-test for continuous variables. In each group, data were compared using the Student's paired *t*-test for continuous variables, between baseline and 1 year later. Absolute changes were measured and presented as variation, defined as the final values minus the initial values. BMD variation was also represented as relative (%) changes. Correlations between changes in BMD and in biochemical parameters were tested using the Pearson correlation coefficient. Several analysis of covariance models for BMD variations (final values minus initial values) were built to analyze the effect of cofactors (continuous variables and discrete variables) on that of infliximab. All analyses were performed with SPSS (SPSS Institute, Cary, NC, USA) software.

## Results

### Control and infliximab group characteristics at baseline

Demographic, clinical, biological and BMD characteristics of the 189 RA patients enrolled in the study are summarized in Table 1. Patients exhibited typical clinical and biological features of RA. The study included 152 women and 37 men (mean ± SD age 52.5 ± 14.2 years). The disease duration was 10.6 ± 9.0 years. A total of 118 patients (62%) were on steroids (mean dose 5.30 ± 5.79 mg/day). The DAS28 score was 5.17 ± 1.07. Thirty-five patients (19%) were on biphosphonates. At baseline, serum PTH and 25-OHD levels were 30.5 ± 17.6 pg/ml and 19.7 ± 10.3 ng/ml, respectively. Serum osteocalcin and serum CTX-I were 19.9 ± 11.8 ng/ml and 446 ± 307 pg/ml, respectively. All of these values were in the normal range. The femoral neck and lumbar BMD values were 0.801 ± 0.159 g/cm^2 ^and 0.911 ± 0.143 g/cm^2^, respectively. According to the BMD values of these patients, 50% had osteopenia and 20% had osteoporosis.

**Table 1:** Clinical, biological and densitometry data at baseline

Parameter	Whole population	Infliximab (*n *= 90)	No infliximab (*n *= 99)	*P*
Age	52.5 ± 14.2	50.9 ± 11.8	53.9 ± 15.9	NS
Women (*n *[%])	152 (80)	74 (82)	78 (79)	NS
Disease duration (years)	10.6 ± 9.0	10.2 ± 9.1	10.9 ± 8.9	NS
Patients on steroids (*n *[%])	118 (62)	56 (62)	62 (63)	NS
Mean dose steroids (mg/day)	5.30 ± 5.79	5.40 ± 5.75	5.26 ± 5.83	NS
DAS28 score	5.17 ± 1.07	5.37 ± 0.94	5.00 ± 1.15	NS
Patients on biphosphonates (*n *[%])	35 (19)	15 (17)	20 (20)	NS
Calcaemia (mmol/l)	2.28 ± 0.13	2.32 ± 0.12	2.24 ± 0.12	NS
Alkaline phosphatase (U/l)	74.6 ± 25.6	77.5 ± 29.5	72.0 ± 21.2	NS
Osteocalcin (ng/ml)	19.9 ± 11.8	18.4 ± 9.7	21.2 ± 13.4	NS
CTX-I (pg/ml)	446 ± 307	482 ± 386	418 ± 227	NS
Parathyroid hormone (pg/ml)	30.5 ± 17.6	28.7 ± 15.4	32.0 ± 19.1	NS
25-hydroxycholecalciferol (ng/ml)	19.7 ± 10.3	18.2 ± 10.1	21.2 ± 10.4	NS
Femoral neck BMD (g/cm^2^)	0.801 ± 0.159	0.807 ± 0.156	0.797 ± 0.162	NS
Lumbar BMD (g/cm^2^)	0.911 ± 0.143	0.930 ± 0.142	0.896 ± 0.142	NS
Patients with neck osteopenia (*n *[%])	96 (51)	47 (52)	49 (50)	NS
Patients with lumbar osteopenia (*n *[%])	93 (49)	40 (45)	53 (54)	NS
Patients with neck osteoporosis (*n *[%])	37 (20)	18 (20)	19 (19)	NS
Patients with lumbar osteoporosis (*n *[%])	37 (19)	15 (17)	23 (23)	NS

Values are shown for each parameter at baseline, expressed as mean ± standard deviation or *n *(%). Data for both groups (case and control) were compared using the unpaired Student's *t*-test for continuous variables and the χ^2 ^test for discrete variables. BMD, bone mineral density; CTX-I, C-terminal cross-linking telopeptide of type I collagen; DAS, Disease Activity Score; NS, not signficant.

When the two groups were compared, no significant variation was observed. In particular, the BMD levels were similar at the femoral neck and at the spine between the groups (Table 1).

### Changes in bone mineral density and markers of bone turnover at 1 year

In the control group, after 1 year of follow up femoral neck BMD decreased from 0.797 ± 0.162 g/cm^2 ^to 0.770 ± 0.162 g/cm^2 ^(-2.5%; *P *< 0.001; Table 2). Similarly, lumbar BMD decreased from 0.896 ± 0.142 g/cm^2 ^to 0.861 ± 0.142 g/cm^2 ^(-3.9%; *P *< 0.001). Regarding markers of bone turnover, the osteocalcin and CTX-I levels remained the same at baseline and after 1 year. The same stability was observed for 25-OHD and PTH levels.

**Table 2:** Bone markers and densitometry data at baseline and 1 year later

Group	Baseline	1 year	*P*
Control			
Osteocalcin	21.2 ± 13.4	20.7 ± 11.5	NS
CTX-I	418 ± 227	391 ± 259	NS
Parathyroid hormone	32.0 ± 19.1	36.0 ± 19.7	NS
25-hydroxycholecalciferol	21.2 ± 10.4	22.8 ± 10.5	NS
Femoral neck BMD	0.797 ± 0.162	0.770 ± 0.162	<0.001
Lumbar BMD	0.896 ± 0.142	0.861 ± 0.142	<0.001
Infliximab			
Osteocalcin	18.4 ± 9.7	15.7 ± 10.9	NS
CTX-I	482 ± 386	452 ± 271	NS
Parathyroid hormone	29.1 ± 15.4	32.6 ± 15.5	NS
25-hydroxycholecalciferol	17.9 ± 10.1	19.6 ± 9.2	NS
Femoral neck BMD	0.807 ± 0.156	0.809 ± 0.151	NS
Lumbar BMD	0.930 ± 0.142	0.928 ± 0.136	NS

Values are shown for each parameter at baseline and at 1 year, expressed as mean ± standard deviation. In each group, data were compared using the Student's paired *t*-test for continuous variables, between baseline and 1 year. BMD, bone mineral density; CTX-I, C-terminal cross-linking telopeptide of type I collagen.

In the infliximab group, femoral and lumbar BMD values exhibited no change between baseline and 1 year. The femoral neck BMD remained stable, being 0.807 ± 0.156 g/cm^2 ^at baseline and 0.809 ± 0.151 g/cm^2 ^at 1 year (+0.2%; not significant). The lumbar BMD was also stable, being 0.930 ± 0.142 g/cm^2 ^at baseline and 0.928 ± 0.136 g/cm^2 ^at 1 year (-0.2%; not significant). Regarding markers of bone turnover, the osteocalcin and CTX-I levels did not change significantly between baseline and 1 year.

### Effect of infliximab on bone mineral density values over 1 year

Various factors are known to influence BMD, including age, sex, menopause status and steroid use. In order to examine the specific effect of infliximab within the context of these possible confounding factors, various linear regression models were used (Table 3). A direct effect of infliximab in a simple model was observed, leading to an increase in BMD values of 0.029 g/cm^2 ^(*P *= 0.001) at the femoral neck and 0.037 g/cm^2 ^(*P *= 0.001) at the lumbar spine (model 1). The effects of infliximab on BMD remained after stratification for male sex and age (model 2). After stratification for male sex, age and steroid use, the effect of infliximab on BMD values at the lumbar spine remained (0.033 g/cm^2^; *P *= 0.001), although the effect was not significant at the femoral neck (0.020 g/cm^2^; *P *= 0.03; model 3). Stratification for male sex, age, menopause status, steroid status and DAS28 level (model 4) did not change the association of infliximab treatment with increased BMD at the lumbar spine (0.034 g/cm^2^; *P *= 0.001) and at the femoral neck (0.023 g/cm^2^; *P *= 0.01). When we added biphosphonate status to the model (model 5), similar effects of infliximab on BMD were observed at the lumbar spine (0.035 g/cm^2^; *P *= 0.001) and at the femoral neck (0.023 g/cm^2^; *P *= 0.02). Infliximab was found to have similar effects on BMD at the lumbar spine (0.041 g/cm^2^; *P *= 0.001) and at the femoral neck (0.028 g/cm^2^; *P *= 0.002) when baseline BMD values were taken into account (Model 6).

**Table 3:** Analysis of the effect of infliximab on BMD using different linear regression models

Models	Cofactors	Femoral neck BMD		Lumbar BMD	
		BMD variation	*P*	BMD variation	*P*

1	Infliximab	0.027	0.001	0.030	0.001
2	Infliximab	0.027	0.001	0.031	0.001
	Male sex	-0.009	0.40	0.008	0.47
	Age	0.002	0.56	0.005	0.09
3	Infliximab	0.020	0.03	0.033	0.001
	Male sex	-0.014	0.22	0.006	0.64
	Age	0.001	0.86	0.005	0.15
	Patients on steroids	-0.012	0.20	-0.001	0.92
4	Infliximab	0.023	0.01	0.034	0.001
	Male	-0.014	0.22	0.006	0.61
	Age	-0.001	0.90	-0.001	0.88
	Menopause	0.005	0.75	0.022	0.20
	Patients on steroids	-0.010	0.29	-0.002	0.87
	DAS28	-0.008	0.04	-0.006	0.17
5	Infliximab	0.023	0.02	0.035	0.001
	Male	-0.015	0.20	0.007	0.58
	Age	-0.001	0.85	0.000	0.95
	Menopause	0.006	0.70	0.020	0.25
	Patients on steroids	-0.009	0.37	-0.004	0.65
	DAS28	-0.008	0.05	-0.006	0.15
	Patients on biphosphonates	-0.012	0.29	0.022	0.07
6	Infliximab	0.028	0.002	0.041	0.001
	Male	-0.007	0.53	0.010	0.39
	Age	-0.002	0.61	0.002	0.63
	Patients on steroids	-0.008	0.35	-0.001	0.91
	DAS28	-0.007	0.06	-0.007	0.10
	Baseline BMD values	-0.042	0.16	-0.080	0.01

All 189 patients were included in several analysis of covariance models for bone mineral density (BMD) variations (final values minus initial values) to analyze the effect of cofactors (continuous variables and discrete variables) on that of infliximab. DAS, Disease Activity Score.

### Relation to infliximab response

**Figure 1:**
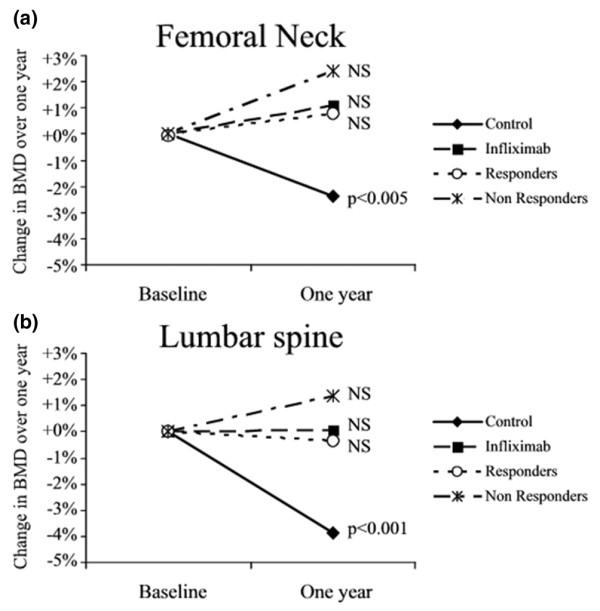
Changes in BMD over 1 year. Changes over 1 year in bone mineral density (BMD), for methotrexate (control) and infliximab groups, are represented in percentages at **(a) **the femoral neck and **(b) **lumbar spine. For the infliximab group, changes in BMD are also separated according to the clinical response, defined by an improvement of at least 1.2 in Disease Activity Score (DAS)28 score over 1 year. NS, not significant.

Sixty-four patients (64%) receiving infliximab were classified as good responders, as defined by an improvement of at least 1.2 in DAS28 score at 1 year. At baseline, no significant difference was observed in BMD at the two sites between clinical responders and nonresponders. The change in lumbar spine BMD was +0.4% (0.890 ± 0.163 g/cm^2 ^at baseline and 0.894 ± 0.154 g/cm^2 ^1 year later) for nonresponders and -0.8% (0.955 ± 0.141 g/cm^2 ^at baseline and 0.947 ± 0.127 g/cm^2 ^1 year later) for responders. Similarly, the change in BMD at the femoral neck was +2.0% (0.765 ± 0.157 g/cm^2 ^at baseline and 0.780 ± 0.174 g/cm^2 ^1 year later) in the nonresponders and -0.4% (0.840 ± 0.142 g/cm^2 ^at baseline and 0.836 ± 0.141 g/cm^2 ^1 year later) in responders (Figure 1). Accordingly, there was no significant difference in BMD between responders and nonresponders. These results indicate that there was bone protection in patients classified as nonresponders, in whom a positive response in terms of RA signs and symptoms could not be demonstrated.

### Correlation between markers of bone turnover and clinical parameters or bone mineral density values

On looking for correlation between bone markers and clinical parameters in the whole population (control and infliximab groups), some interesting correlations were observed. Negative correlations were observed between age and BMD at the lumbar spine and femoral neck (*r *= -0.279, *P *< 0.001 and *r *= -0.398, *P *< 0.001, respectively). Steroid dose correlated with lower levels of bone formation markers, reflected by osteocalcin level (*r *= -0.322; *P *< 0.001). Similarly, when we looked for correlation between BMD values and markers of bone turnover in the whole population, we observed a negative correlation between osteocalcin levels and lumbar spine and femoral neck BMD (*r *= -0.301, *P *< 0.001 and *r *= -0.193, *P *< 0.001, respectively). However, we observed no association between changes in bone markers and changes in BMD values.

## Discussion

To our knowledge, this appears to be the first study to examine the effect of infliximab on BMD in RA patients and to compare the findings with those in a control group. The major finding of our study is that bone loss was prevented in RA patients treated with infliximab. This protective effect was also observed in patients who did not exhibit a clinical response.

Bone loss in RA patients depends on a number of factors. This patient population is already at high risk for osteoporosis because of advanced age, female sex and menopause. In addition, active disease and extended duration of disease also have indirect adverse effects on bone, combined with the use of steroids and even methotrexate. Selecting an appropriate control group for such a study is difficult. Even a planned placebo-controlled trial is imperfect, because the application of exclusion criteria represents a difference from the real world situation. Accordingly, we opted to use historical controls because infliximab is now on the market, and it would be unethical not to use and even to withhold infliximab treatment from RA patients with active disease. We regarded the optimal situation to be one in which the only major difference between groups would be receipt/nonreceipt of infliximab. Outside the setting of a formal clinical trial, the best available option was to select a population of RA patients followed at the same institution at a time before the advent of anti-TNF-α treatment.

We first verified that our populations at baseline were representative. We were able to confirm the presence of the typical correlations of age and disease duration with lower BMD. Similarly, patients receiving steroids exhibited lower levels of markers of bone formation markers. Our findings of these usual correlations provide validation of the data at baseline.

In the study, we were unable to demonstrate an increase in BMD with infliximab, as was recently observed in smaller studies conducted without a control group in RA patients (*n *= 26) [9] and in patients with spondylarthropathy (*n *= 29) [14] and Crohn's disease (*n *= 46) [15]. Recently, however, infliximab was found to prevent deterioration in BMD at spine and hip, but not in hands in RA patients [10]. Once again, however, no control group was employed.

Regarding markers of bone turnover, no significant changes were observed over the 1 year of follow up. However, negative correlations between BMD and osteocalcin in this population contrast with the physiological association between osteocalcin and bone formation. The findings suggest a defect in bone formation that may be explained, in part, by systemic inflammation. Indeed, in one of our previous studies [16], uncoupling between bone destruction and formation was observed in destructive RA. Such uncoupling was not observed in benign RA. It now appears that TNF-a inhibition may restore this coupling of destruction with formation.

Early changes in bone markers were previously observed in a study conducted in 68 RA patients who were treated with infliximab [17]. Bone formation markers (osteocalcin and N-propeptide of type I procollagen) increased quickly at weeks 2 and 6 after initiation of infliximab treatment. The long-term effect of infliximab at 1 year was recently described in a small population of patients with RA (*n *= 26) [9], manifesting as a persistent increase in osteocalcin (reflecting bone formation) and a persistent decrease in CTX-I (reflecting bone resorption). However, in 70 patients with RA who were treated with infliximab plus methotrexate, a decrease was observed only in bone resorption markers (urinary excretion of N-telopeptide of type I collagen and deoxypyridinoline) [18]. No effect on serum bone alkaline phosphatase, a marker of bone formation, was observed. In patients with spondylarthropathy treated with infliximab, an early decrease in CTX-I was observed at 3 months (-50%; *P *= 0.005). However, CTX-I levels further increased at 1 year as compared with baseline [19]. In another study, infliximab therapy had a lesser effect on serum osteoprotegerin and soluble receptor activator of nuclear factor-kB ligand (sRANKL) in RA patients [20]. Recently, decreases in CTX-I and sRANKL were observed over 1 year of infliximab treatment [10]. However, in none of these studies was a control group included, preventing a true estimation of the effect of anti-TNF-a treatment on bone. In this study, an early and transient effect on markers of bone turnover (increased bone formation and decreased bone resorption) was found to be associated with the effect on BMD at 1 year, as was demonstrated in a previous study for CTX-I [10].

It appears that blocking TNF-a is protective with respect to bone mass, and this effect of infliximab on BMD was independent of use of biphosphonates and other putative covariates. That this protective effect was also observed in clinical nonresponders to infliximab is in accordance with the findings of a recent study [8] that demonstrated protective effects on joint destruction in the absence of clinical response. Similarly, studies conducted in postmenopausal women [4] identified an association between activated status in blood mononuclear cells/monocytes and increased production of proinflammatory cytokines. This increase was sensitive to oestrogen replacement.

## Conclusion

This study indicates that TNF-a inhibition controls bone loss related to generalized osteoporosis within the context of RA. This is another reason to differentiate local joint inflammation from juxta-articular bone destruction.

## Abbreviations

BMD = bone mineral density; CTX-I = C-terminal cross-linking telopeptide of type I collagen; DAS = Disease Activity Score; 25-OHD = 25-hydroxycholecalciferol; PTH = parathyroid hormone; RA = rheumatoid arthritis; SD = standard deviation; sRANKL = soluble receptor activator of nuclear factor-kB ligand; TNF = tumour necrosis factor.

## Competing interests

The authors declare that they have no competing interests.

## Authors' contributions

The initial draft of the report was written by HM and PM. Patients were recruited and clinical parameters provided by HM, BP-P, LG, PG, CA and PM. The paper was written by HM and PM.
